# Supplementary material for: Structural and functional characterization of the bacterial biofilm activator RemA
Source: Nat Commun. 2021 Sep 29;12:5707. doi: 10.1038/s41467-021-26005-4 (PMC8481266; doi:10.1038/s41467-021-26005-4)
Supplement: Supplementary file 1 — Supplementary Information [file 41467_2021_26005_MOESM1_ESM.pdf]

Supplementary information for:

Structural and functional characterization of the bacterial biofilm activator RemA

Tamara Hoffmann[1,5], Devid Mrusek[2,5], Patricia Bedrunka[2,5], Fabiana Burchert[2], Christopher Nils Mais[2], Daniel B. Kearns[3], Florian Altegoer[2], Erhard Bremer[1,6] and Gert Bange[2,4,6]

[1]Philipps-University Marburg, Center for Synthetic Microbiology (SYNMIKRO) & Faculty of Biology, Karl-von-Frisch Strasse 14, 35043 Marburg, Germany

[2]Philipps-University Marburg, Center for Synthetic Microbiology (SYNMIKRO) & Faculty of Chemistry, Karl-von-Frisch Strasse 14, 35043 Marburg, Germany

[3]Department of Biology, Indiana University, Bloomington IN 47405.

[4]Max Planck Institute for Terrestrial Microbiology, Karl-von-Frisch Strasse 10, 35043 Marburg, Germany

[5]These authors contributed equally to this work.

[6]Correspondence:

gert.bange@synmikro.uni-marburg.de; bremer@staff.Uni-Marburg.de

Supplementary Figures 1 – 5

Supplementary Tables 1 – 4

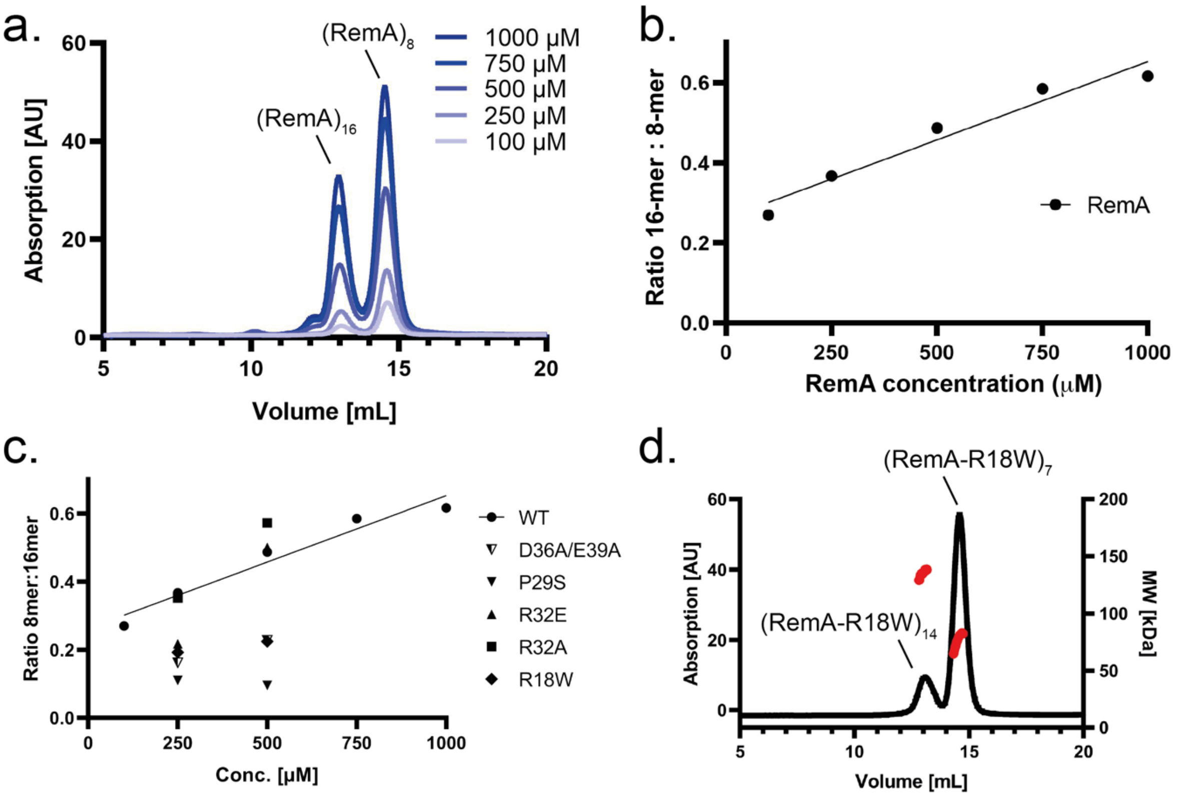

Supplementary Figure 1. RemA forms 8- and 16-mers in a concentration-dependent manner a. Chromatograms of size exclusion chromatography of RemA at different concentrations as indicated in the figure. The wavelength was 278 nm. **b.** The (*Gt*)$(RemA)_8/(RemA)_{16}$ ratio as shown in supplementary figure 1a is plotted according to the RemA concentration injected to the SEC. Line represents a linear fit (R=0,9514). Each data point represents an independent size exclusion run. **c.** The $(RemA)_8/(RemA)_{16}$ ratio for wildtype RemA and different RemA variants at different protein concentration is shown. **d.** Chromatogram of an analytical SEC (black lines)-MALS (red lines) experiment of RemA-R18W. Source data are provided as a Source data file.

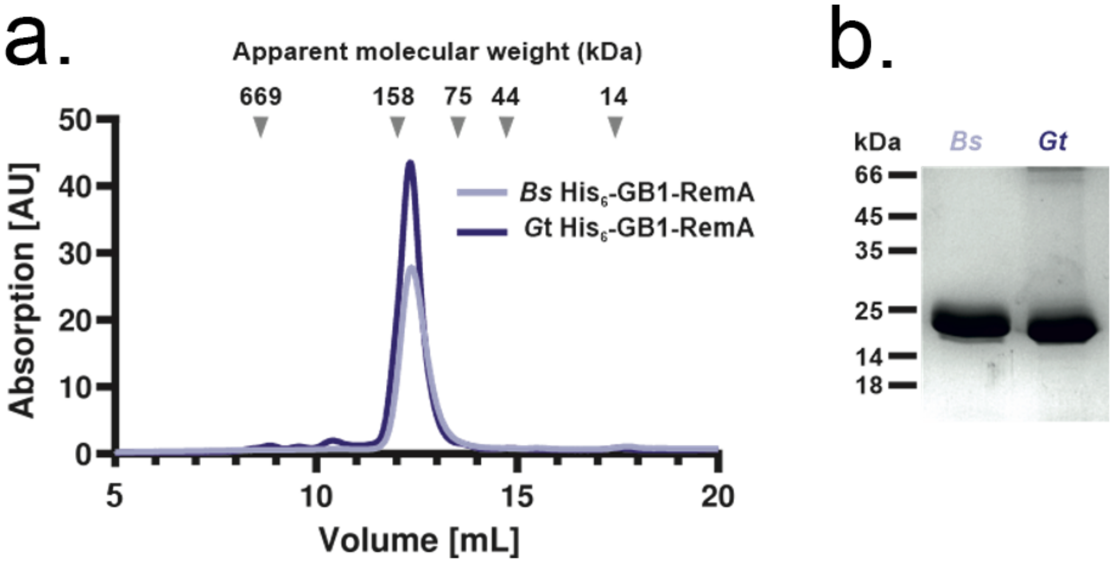

Supplementary Figure 2. Size exclusion chromatograms of His$_6$-GB1 fusions of (*Bs*)RemA and (*Gt*)RemA. a. Analytical size-exclusion chromatograms (as measured at a wavelength of 278 nm) of His$_6$-GB1-(*Bs*)RemA and of His$_6$-GB1-(*Gt*)RemA. Arrows indicate elution volumes and mass of molecular weight standards. The expected molecular weight for His$_6$-GB1-(*Bs*)RemA is 136 kDa; the estimated molecular weight for His$_6$-GB1-(*Gt*)RemA is 138 kDa. **b**. Coomassie-stained SDS-PAGE of His$_6$-GB1-(*Bs*)RemA and of His$_6$-GB1-(*Gt*)RemA (main peak fraction) after preparative size-exclusion chromatography. At least three independent size exclusion runs confirmed the presence of octameric RemA fusion proteins. Both monomeric fusion proteins have a theoretical molecular weight of approximately 20 kDa. Source data are provided as a Source data file.

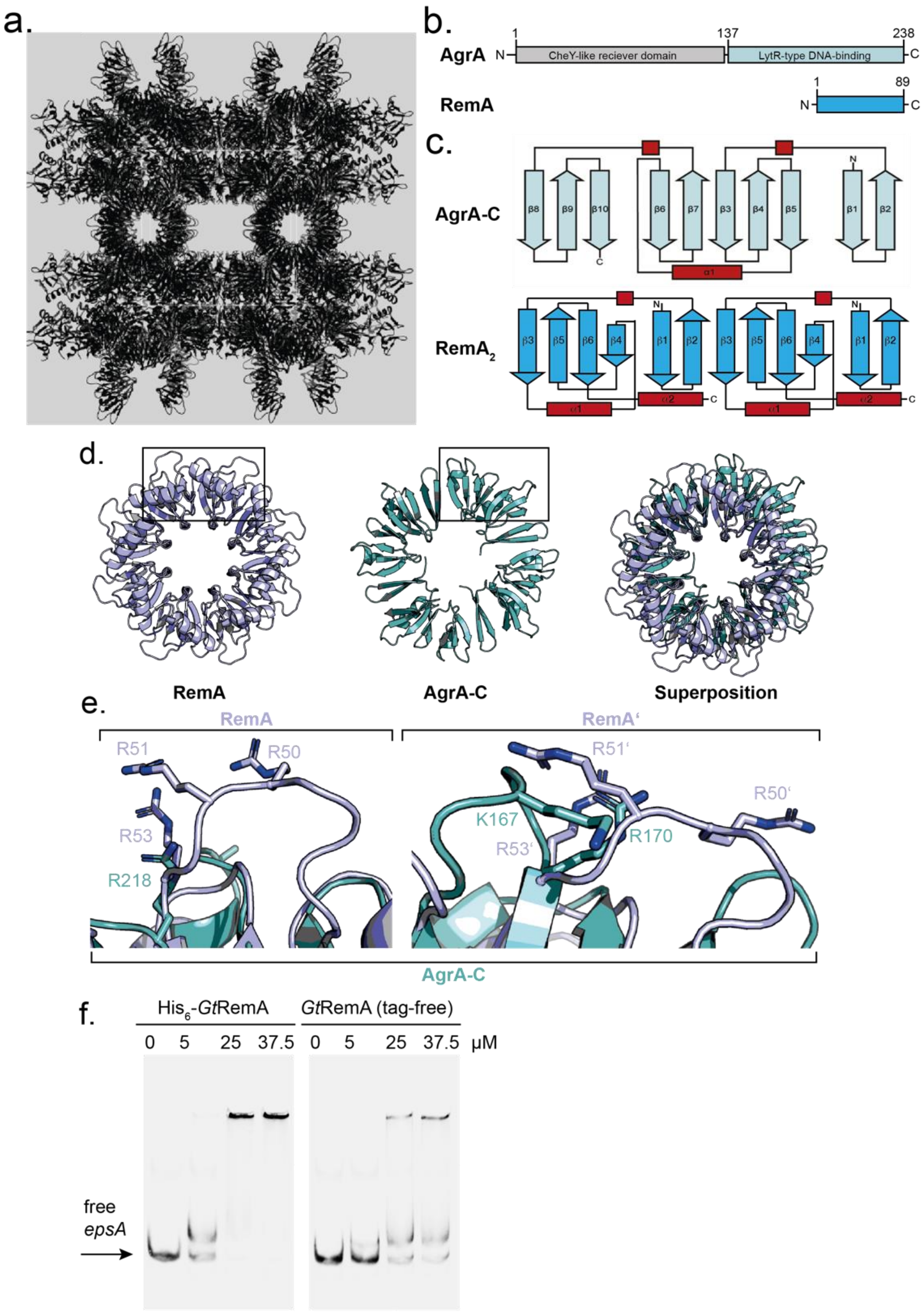

Supplementary Figure 3. Structure of (*Gt*)RemA. a. Crystal lattice of (*Gt*)RemA. The unit cell is shown as white lines. **b.** Domain overview of AgrA and RemA. **c.** Schematic representation of the secondary/ternary structure arrangement of AgrA-C and $(RemA)_2$ shows that AgrA-C is highly reminiscent to a RemA dimer. Blue arrows and red boxes represent β-strands and α-helices, respectively. 'N' and 'C' indicates N- and C-termini, respectively. AgrA-C contains all secondary structural elements of RemA, except for α1 and β4. **d.** The RemA octamer (left, PDB-ID: 7BM2) is highly reminiscent of the AgrA-C tetramer (middle, PDB-ID: 3BS1) as shown by a superimposition (right) of the ring assemblies. **e.** Superimposition of RemA (light blue) and AgrA-C (pale green) shows that positive residues decorating the outer ring are in similar positions. **f.** EMSA of wild type (*Gt*)RemA proteins with or without a N-terminal hexa-histidine tag with a DNA fragment containing the regulatory *epsA* region. Results were confirmed with three independent preparations of recombinant protein. Source data are provided as a Source data file.

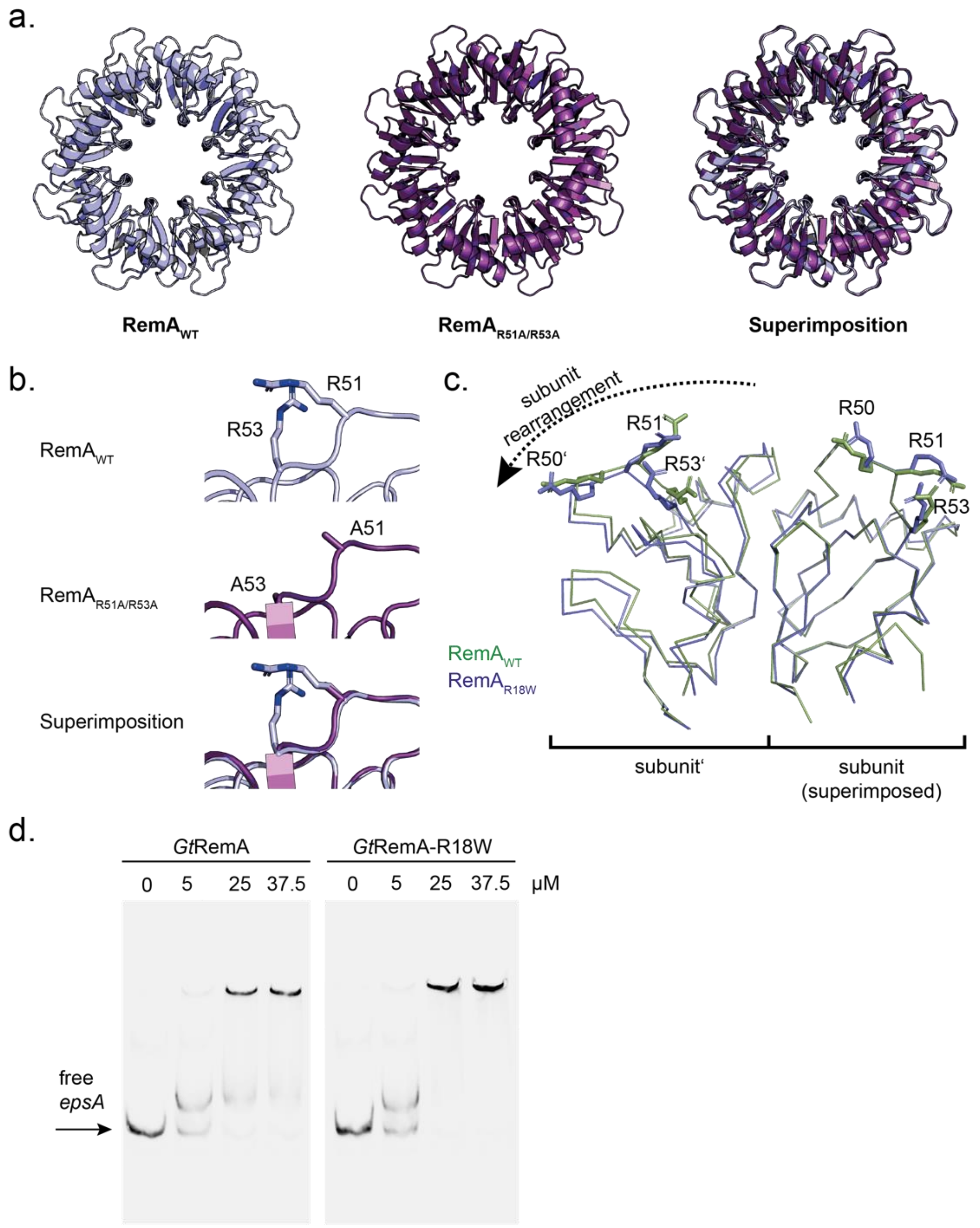

Supplementary Figure 4. a. Superimposition of $(RemA)_8$ and $(RemA_{R51AR53A})_8$ shows no deviations between the two assemblies. **b.** Superimposition of RemA (light blue, PDB-ID: 7BM2) wildtype and $RemA_{R51AR53A}$ (purple, PDB-ID: 7P1W) shows that both proteins superpose well with a r.m.s.d. of 0.23 while positioning of residue 51 and 53 is unaffected **c.** Superimposition of two subunits of $(RemA)_8$ (green) and $(RemA\text{-}R18W)_7$ (blue) on one subunit (indicated as "superimposed") shows the relative rearrangement of the subunits to each other, which also leads to a repositioning of the DNA-binding arginines 50, 51 and 53 at the surface. **d.** EMSA of wild type (*Gt*)RemA and its R18W variant with a DNA fragment containing the regulatory *epsA* region. Results were confirmed with at least three independent preparations of recombinant protein. Source data are provided as a Source data file.

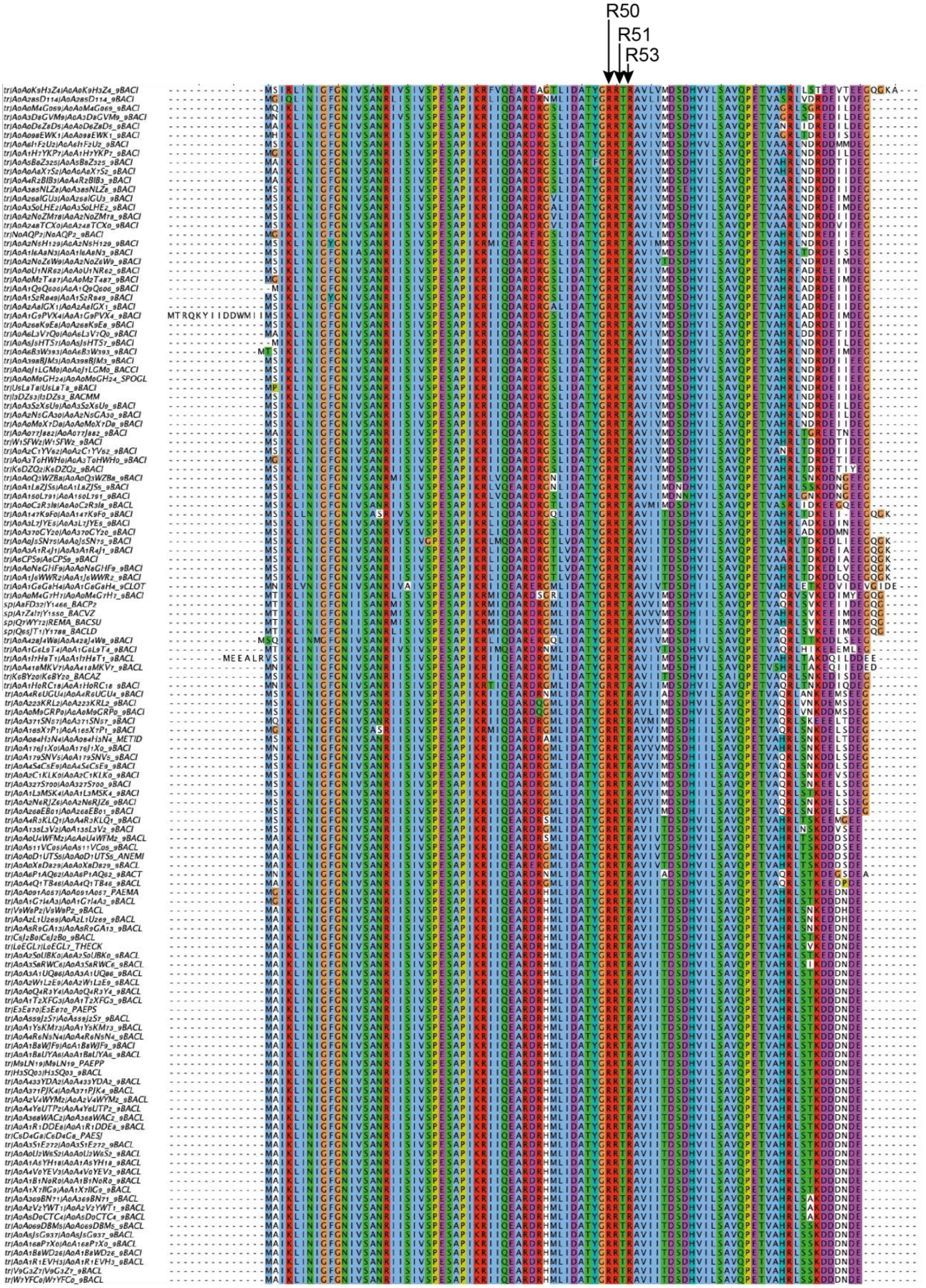

Supplementary Figure 5. Sequence alignment of various RemA proteins from Bacilli shows the arginines 50, 51 and 53, which are essential for DNA binding, are conserved.

Supplementary Table 1: Data collection and refinement statistics

	*Gt*RemA (7BM2)	*Gt*RemA$_{R18W}$ (7BME)	*Gt*RemA$_{R51AR53A}$ (7P1W)
**Data collection**			
Space group	*P*23	*C*222$_1$	*I* 4 2 2
Cell dimensions			
a, *b*, *c* (Å)	106.91 106.91 106.91	103.89 116.97 114.135	89.323 89.323 109.236
α, β, γ (°)	90 90 90	90 90 90	90 90 90
Resolution (Å)	37.8 - 2.291 (2.373 - 2.291)	47.28 - 2.6 (2.693 - 2.6)	37.52 - 1.8 (1.864 - 1.8)
R_{merge}	0.1947 (3.529)	0.1129 (1.786)	0.02446 (0.8002)
$I / \sigma I$	19.48 (1.18)	9.10 (0.79)	13.10 (0.81)
Completeness (%)	99.69 (98.25)	99.58 (99.77)	99.88 (99.76)
Redundancy	36.8 (23.1)	7.2 (7.5)	2.0 (2.0)
$CC_{1/2}$	0.999 (0.562)	0.996 (0.404)	1 (0.49)
Wavelength (Å)	0.969	0.873	0.987
**Refinement**			
Resolution (Å)	37.8 – 2.29	47.28 – 2.60	37.52 – 1.80
No. reflections	18622 (1793)	21692 (2143)	20817 (2043)
R_{work} / R_{free}	17.8/22.3	21.6/27.6	20.0/22.0
No. atoms			
Protein	2425	4044	1172
Ligand/ion	215	0	0
Water	89	7	79
B-factors			
Protein	49.23	88.20	40.48
Ligand/ion	69.55	0	0
Water	50.24	63.69	41.07
Ramachandran (%)			
favored	96.41	96.50	98.01
allowed	3.27	3.50	1.99
outliers	0.33	0.00	0.00
R.m.s. deviations			
Bond lengths (Å)	0.010	0.008	0.004
Bond angles (°)	1.34	1.09	0.79

*Values in parentheses are for highest-resolution shell.

Supplementary Table 2. Plasmids and primers used in the study

Plasmid	Plasmid description	Primers used for construction[a] (sequence 5`→ 3`direction)
pDR111	*amyE*::P_{hy}, spc^R ectopic integration vector with a strong IPTG-inducible promoter	kind gift of D. Rudner (Boston, MA, US)
pBB284	´*ytnM-ytoI*`::$spc^R$ ectopic vector for integration into the ´*ytnM-ytoI*` intergenic region	kind gift of D. Rudner (Boston, MA, US)
pDG268	*amyE*::*lacZ*, cml^R ectopic integration vector with a promoter-less *lacZ* gene	Antoniewski *et al*, 1990 [1]
pFC1	pDG268 P_{epsA}-*lacZ*, cml^R	7539: 5´- AGGAGGAATTCTTGTACGGCTTGCACTAAATGTAC<br>3025: 5´- CTCCTGGATCCATTCATAGCCTTCAGCCTTCCCG
pTMB33	pDR111 (RBS)*remA*	remA-RBS-for: 5´-ATTAAGCTTGAGACGTCTATTTTACAGGGGGA<br>remA-rev: 5`-GCGGCTAGCCCCTCTTTCTTTCATGCGGC
pTMB42	pBB284 (*lacI*-P_{hy} $remA^{wild\ type}$, spc^R)1	
pTMB87	pBB284 (*lacI*-P_{hy} $remA^{R32A}$, spc^R)4	Bs-remA-R32A-for: 5` -GCCAATCAAAgcGATGATTCAGGATGCAAGAGACCG<br>Bs-remA-R32-rev: 5`-GCAGACTCCGGGCTGACA
pTMB88	pBB284 (*lacI*-P_{hy} $remA^{D36S}$, spc^R)5	Bs-remA-D36S-for: 5`-GATGATTCAGtcTGCAAGAGACCGCG<br>Bs-remA-D36S-rev: 5` -CGTTTGATTGGCGCAGAC
pTMB90	pBB284 (*lacI*-P_{hy} $remA^{D39K}$, spc^R)7	Bs-remA-D39K-for: 5`-GGATGCAAGAaagCGCGGAATGC<br>Bs-remA-D39K-rev: 5`-CCTGAATCATCCGTTTGATTGG
pTMB92	pBB284 (*lacI*-P_{hy} $remA^{R18W}$, spc^R)9	Bs-remA-R18W-for: 5`-CTCCGCCAATtGGATGATTTC<br>Bs-remA-R18W-rev: 5`-ATGATATTGCCAAATCCG
pTMB93	pBB284 (*lacI*-P_{hy} $remA^{P29S}$, spc^R)10	Bs-remA-P29S-for: 5`-GGAGTCTGCGtCAATCAAACG<br>Bs-remA-P29S-rev: 5`-GGGCTGACAATCGAAATC
pTMB94	pBB284 (*lacI*-P_{hy} $remA^{R50A}$, spc^R)11	Bs-remA-R50A-for: 5`-TACATACGGAgcAAGAACCCGTGC<br>Bs-remA-R50A-rev: 5`-GCGTCAATTAGCATTCCG
pTMB95	pBB284 (*lacI*-P_{hy} $remA^{R51A}$, spc^R)12	Bs-remA-R51A-for: 5`-ATACGGACGAgcAACCCGTGCA<br>Bs-remA-R51A-rev: 5`-GTAGCGTCAATTAGCATTC
pTMB109	pBB284 (*lacI*-P_{hy} $remA^{D36A/D39A}$, spc^R)16	155-Q5-remA-D36A-D39A-f: 5`-ATGATTCAGGcTGCAAGAGCCC<br>156-Q5-remA-D36A-D39A-r: 5`-CCGTTTGATTGGCGCAGA
	sinR::spc^R LFR fusion PCR	403: 5´-AATCACTTTTATTACAGATAAAGAAAATG<br>404: 5´-ATCACCTCAAATGGTTCGCTGGGTTTATCAATGTCATCAC<br>405: 5´-AAGTTCGCTAGATAGGGGTCCCGAGCAAGAGGAGTCGT<br>406: 5´-AAAGACAAAAGCCTTGGAAACAGATA
	remA::zeo^R LFR fusion PCR	remA-P1-for: 5´-GCTATATTTTGAAGATAAGCTGAAACAGAC<br>remA-P2-zeo(anti)-rev: 5´-CCATATCAAGATAACTTCGTATAATGTATGTAATCAGTTTAATCGTCATCTTCTACGTTC<br>remA-P3-zeo(anti)-for: 5´-GGACTGAATAACTTCGTATAGCATACATTACTGTTAAAGAAGAAATTATGGATGAAGGGC<br>remA-P4-rev: 5´-TTCTTCTAAAGCTTTGCCTACATATTTGTG

Amplification of the *zeo*^{R} resistance cassette	remA-RC-P2-zeo(anti): 5´-GAACGTAGAAGATGACGATTAAACTGATTACATACATTATACGAAGTTATCTTGATATGG<br>remA-RC-P3-zeo(anti): 5´-GCCCTTCATCCATAATTTCTTCTTTAACAGTAATGTATGCTATACGAAGTTATTCAGTCC
remA::*tet*^{R} LFR fusion PCR	1087: 5´-TAGCGTGTCTATTGCCCTTTTATTAT<br>1088: 5´-CAATTCGCCCTATAGTGAGTCGTAATCAGTTTAATCGTCATCTTCTACG<br>1089: 5´-CCAGCTTTTGTTCCCTTTAGTGAGCAGACTTTCTGTTAAAGAAGAAATTATG<br>1090: 5´-CAGCGATGCCTCCACTCACGCA
289 bp P*epsA* fragment for electrophoretic mobility shift assay (fluorescence label Dyomics 781)	150-epsA(+234)for: 5`-CTCCTCTATTCCTGTCGTTATTTCG<br>149-epsA(-55)rev: 5`-CGAATCTGTGTCTGACAATCGC

[a] Blue color indicates the ribosome binding site of *remA*. Red color indicates codons changed in *remA.*

Supplementary Table 3. ***B. subtilis*** **strains used in this study**

Strain	Genotype or description	Reference, source or construction[a]
PY79	wild type	Youngman *et al*, 1984 [2]
BDR2258	PY79 ´*ytnM-ytoI*`::*cml*^{R} marker-replacement strain	kind gift of D. Rudner (Boston, MA, US)
TMB410	JH642 ´*ytnM-ytoI*`::*cml*^{R} marker-replacement strain	chromosomal DNA (BDR2258) → → JH642
TMB413	JH642 (´*ytnM-ytoI*`::*lacI*-P$_{hy}$ *remA*$^{wild\ type}$, *spc*^{R})1	linearized (PvuI) pTMB42 → TMB410
TMB489	JH642 (´*ytnM-ytoI*`::*lacI*-P$_{hy}$ *remA*^{R32A}, *spc*^{R})4	linearized (PvuI) pTMB87 → TMB410
TMB491	JH642 (´*ytnM-ytoI*`::*lacI*-P$_{hy}$ *remA*^{D36S}, *spc*^{R})5	linearized (PvuI) pTMB88 → TMB410
TMB495	JH642 (´*ytnM-ytoI*`::*lacI*-P$_{hy}$ *remA*^{D39K}, *spc*^{R})7	linearized (PvuI) pTMB90 → TMB410
TMB499	JH642 (´*ytnM-ytoI*`::*lacI*-P$_{hy}$ *remA*^{R18W}, *spc*^{R})9	linearized (PvuI) pTMB92 → TMB410
TMB501	JH642 (´*ytnM-ytoI*`::*lacI*-P$_{hy}$ *remA*^{P29S}, *spc*^{R})10	linearized (PvuI) pTMB93 → TMB410
TMB503	JH642 (´*ytnM-ytoI*`::*lacI*-P$_{hy}$ *remA*^{R50A}, *spc*^{R})11	linearized (PvuI) pTMB94 → TMB410
TMB505	JH642 (´*ytnM-ytoI*`::*lacI*-P$_{hy}$ *remA*^{R51A}, *spc*^{R})12	linearized (PvuI) pTMB95 → TMB410
TMB561	JH642 (´*ytnM-ytoI*`::*lacI*-P$_{hy}$ *remA*$^{D36A/D39A}$, *spc*^{R})16	linearized (PvuI) pTMB109 → TMB410
P*epsA*-reporter strains		
JH642	*pheA*1 *trpC*2 wild type	J. Hoch; BGSC[b] (1A96)
TMB196	JH642 (*remA*::*zeo*^{R})1	LFR PCR (*remA*´-zeoR-`*remA*) → JH642
DS859	NCIB3610 (*sinR*::*kan*^{R})	LFR PCR (*sinR*´-kanR-`*sinR*) → PY79 → NCIB3610
DS518	NCIB3610 *amyE*::P*epsA-lacZ, cml*^{R}	linearized pFC1 → PY79 → NCIB3610
TMB523	JH642 (*remA*::*zeo*^{R})1 *sinR*::*kan*^{R}	chromosomal DNA (DS859) → TMB196
TMB524	JH642 (*remA*::*zeo*^{R})1 *sinR*::*kan*^{R} *amyE*::P*epsA-lacZ, cml*^{R}	chromosomal DNA (DS518) → TMB523
TMB532	JH642 (*remA*::*zeo*^{R})1 *sinR*::*kan*^{R} *amyE*::P*epsA-lacZ, cml*^{R} (´*ytnM-ytoI*`::*lacI*-P$_{hy}$ *remA*$^{wild\ type}$, *spc*^{R})1	chromosomal DNA (TMB413) → TMB523
TMB536	JH642 (*remA*::*zeo*^{R})1 *sinR*::*kan*^{R} *amyE*::P*epsA-lacZ, cml*^{R} (´*ytnM-ytoI*`::*lacI*-P$_{hy}$ *remA*^{R32A}, *spc*^{R})4	chromosomal DNA (TMB489) → TMB523

TMB537	JH642 (*remA*::*zeo*^{R})1 *sinR*::*kan*^{R} *amyE*::P*epsA-lacZ, cmI*^{R} (*´ytnM-ytoI`*::*lacI*-P$_{hy}$ *remA*^{D36S}, *spc*^{R})5	chromosomal DNA (TMB491) → TMB523
TMB540	JH642 (*remA*::*zeo*^{R})1 *sinR*::*kan*^{R} *amyE*::P*epsA-lacZ, cmI*^{R} (*´ytnM-ytoI`*::*lacI*-P$_{hy}$ *remA*^{D39K}, *spc*^{R})7	chromosomal DNA (TMB495) → TMB523
TMB541	JH642 (*remA*::*zeo*^{R})1 *sinR*::*kan*^{R} *amyE*::P*epsA-lacZ, cmI*^{R} (*´ytnM-ytoI`*::*lacI*-P$_{hy}$ *remA*^{R18W}, *spc*^{R})9	chromosomal DNA (TMB499) → TMB523
TMB542	JH642 (*remA*::*zeo*^{R})1 *sinR*::*kan*^{R} *amyE*::P*epsA-lacZ, cmI*^{R} (*´ytnM-ytoI`*::*lacI*-P$_{hy}$ *remA*^{P29S}, *spc*^{R})10	chromosomal DNA (TMB501) → TMB523
TMB543	JH642 (*remA*::*zeo*^{R})1 *sinR*::*kan*^{R} *amyE*::P*epsA-lacZ, cmI*^{R} (*´ytnM-ytoI`*::*lacI*-P$_{hy}$ *remA*^{R50A}, *spc*^{R})11	chromosomal DNA (TMB503) → TMB523
TMB544	JH642 (*remA*::*zeo*^{R})1 *sinR*::*kan*^{R} *amyE*::P*epsA-lacZ, cmI*^{R} (*´ytnM-ytoI`*::*lacI*-P$_{hy}$ *remA*^{R51A}, *spc*^{R})12	chromosomal DNA (TMB505) → TMB523
TMB565	JH642 (*remA*::*zeo*^{R})1 *sinR*::*kan*^{R} *amyE*::P*epsA-lacZ, cmI*^{R} (*´ytnM-ytoI`*::*lacI*-P$_{hy}$ *remA*$^{D36A/D39A}$, *spc*^{R})16	chromosomal DNA (TMB561) → TMB523
Biofilm strains		
DK1042	*comI*^{Q12L}	Konkol *et al*., 2013 [3]
DK7212	*comI*^{Q12L} (*remA*::*tet*^{R})3	LFR PCR (*remA´*-tetR-*`remA*) → DK1042
DK6673	*comI*^{Q12L} (*remA*::*tet*^{R})3 (*´ytnM-ytoI`*::*lacI*-P$_{hy}$ *remA*^{R32A}, *spc*^{R})4	linearized (PvuI) pTMB87 → DK1042
DK6674	*comI*^{Q12L} (*remA*::*tet*^{R})3 (*´ytnM-ytoI`*::*lacI*-P$_{hy}$ *remA*^{P29S}, *spc*^{R})10	linearized (PvuI) pTMB93 → DK1042
DK6675	*comI*^{Q12L} (*remA*::*tet*^{R})3 (*´ytnM-ytoI`*::*lacI*-P$_{hy}$ *remA*^{R50A}, *spc*^{R})11	linearized (PvuI) pTMB94 → DK1042
DK6847	*comI*^{Q12L} (*remA*::*tet*^{R})3 (*´ytnM-ytoI`*::*lacI*-P$_{hy}$ *remA*$^{wild\ type}$, *spc*^{R})1	linearized (PvuI) pTMB42 → DK1042
DK6849	*comI*^{Q12L} (*remA*::*tet*^{R})3 (*´ytnM-ytoI`*::*lacI*-P$_{hy}$ *remA*^{D36S}, *spc*^{R})5	linearized (PvuI) pTMB88 → DK1042
DK7215	*comI*^{Q12L} (*remA*::*tet*^{R})3 (*´ytnM-ytoI`*::*lacI*-P$_{hy}$ *remA*^{R18W}, *spc*^{R})9	linearized (PvuI) pTMB92 → DK1042
DK7216	*comI*^{Q12L} (*remA*::*tet*^{R})3 (*´ytnM-ytoI`*::*lacI*-P$_{hy}$ *remA*^{R51A}, *spc*^{R})12	linearized (PvuI) pTMB95 → DK1042
TMB593	*comI*^{Q12L} (*remA*::*tet*^{R})3 (*´ytnM-ytoI`*::*lacI*-P$_{hy}$ *remA*^{D39K}, *spc*^{R})7	SPP1 Lysate of TMB495 → DK7212
TMB594	*comI*^{Q12L} (*remA*::*tet*^{R})3 (*´ytnM-ytoI`*::*lacI*-P$_{hy}$ *remA*$^{D36A/D39A}$, *spc*^{R})16	SPP1 Lysate of TMB561 → DK7212

[a] Linearized plasmid, PCR product or genomic DNA (left side of the arrow; name or origin given in parenthesis) used to transform an existing strain (right side of the arrow) used for construction. SPP1 phage lysates of a *B. subtilis* strain (given in parentheses) used to transduce a given allele into an existing strain (right side of the arrow) to create a new strain are listed.

[b] BGSC: Bacillus Genetic Stock Center (Columbus, OH, USA)

Supplementary Table 4.

Plasmid	Plasmid description	Primers used for construction[a] (sequence 5`→ 3`direction)
pDM243	pET24d N-6H-(*Gt)remA*	Gt-remA-6H-for: 5’-TTAACCATGGGCCATCACCATCACCATCACATGATGAAGTTTATTAATATCG<br>Gt-remA-rev: 5’-TTAACTCGAGTTACCCTTCCTCGGAGAAATC
pDM286	pET24d N-6H-(*Gt)remA*	Gt-remA-P29S-for: 5’-CGGCGTCGATTAAACGAATC<br>Gt-remA-P29S-rev: 5’-TTCGCGCGCATCTTGGATGATTCGTTTAATCGACGCCG
pDM288	pET24d N-6H-(*Gt)remA*	Gt-remA-R18W-for: 5’-GCCGCCTGGATCATTACGATT<br>Gt-remA-R18W-rev: 5’-GCCGAATCGGGGCTGACAATCGTAATGATCCAGGCGGC
pDM292	pET24d N-6H-(*Gt)remA*	Gt-remA-R32A-for: 5’-CCGATTAAAGCAATCATCCAAGATGCGC<br>Gt-remA-R32A-rev: 5’-CGACGAGCTTACCTTTTTCGCGCGCATCTTGGATGATTGCTTTAATCG
pAL108	pET24d N-6H-(*Gt)remA*	Gt-remA-R32E-for: 5’-ccgattaaagaaatcatccaagatgcgc<br>Gt-remA-R32E-rev: 5’-GCGCATCTTGGATGATTAGTTTAATCGG
pAL111	pET24d N-6H-(*Gt)remA*	Gt-remA-D36A-E39A-for: 5’-CATCCAAGCTGCGCGCGCAAAAGGTAAGCTCGTCG<br>Gt-remA-D36A-E39A-rev: 5’-CGACGAGCTTACCTTTTGCGCGCGCAGCTTGGATG
pPB166	pET-N-6H-GB1-(*Gt)remA*	PB158fwd: 5´-TTAAGGTCTCCCATGGGCATGATGAAGTTTATTAATATCGGATACGG<br>PB158rev: 5´-TTAAGGTCTCCTCGAGTTACCCTTCCTCGGAGAAATCATC
pPB173	pET-N-6H-GB1-(*Bs)remA*	PB156fwd: 5´-TTAAGGTCTCCCATGGGCACGATTAAACTGATTAATATCGGATTTGG<br>PB155rev: 5´-TTAAGGTCTCCTCGAGTTACCCCTGCCCTTCATCC

Supplementary References:

1. Antoniewski, C., Savelli, B. & Stragier, P. The *spoIIJ gene*, which regulates early developmental steps in *Bacillus subtilis*, belongs to a class of environmentally responsive genes. *J. Bacteriol.* **172**, 86–93 (1990).
2. Youngman, P., Perkins, J. B. & Losick, R. Construction of a cloning site near one end of Tn917 into which foreign DNA may be inserted without affecting transposition in *Bacillus subtilis* or expression of the transposon-borne erm gene. *Plasmid* **12**, 1–9 (1984).
3. Konkol, M. A., Blair, K. M. & Kearns, D. B. Plasmid-encoded *comI* inhibits competence in the ancestral 3610 strain of *Bacillus subtilis*. *J. Bacteriol.* **195**, 4085–4093 (2013).
