# Supplementary material for: Structural and functional characterization of the bacterial biofilm activator RemA
Source: Nat Commun. 2021 Sep 29;12:5707. doi: 10.1038/s41467-021-26005-4 (PMC8481266; doi:10.1038/s41467-021-26005-4)
Supplement: Supplementary file 3 — Reporting Summary [file 41467_2021_26005_MOESM3_ESM.pdf]

nature portfolio

Corresponding author(s): Dr. Gert Bange, Dr. Erhard Bremer

Last updated by author(s): Aug 25, 2021

# Reporting Summary

Nature Portfolio wishes to improve the reproducibility of the work that we publish. This form provides structure for consistency and transparency in reporting. For further information on Nature Portfolio policies, see our Editorial Policies and the Editorial Policy Checklist.

## Statistics

For all statistical analyses, confirm that the following items are present in the figure legend, table legend, main text, or Methods section.

| n/a | Confirmed | |
|---|---|---|
| ☐ | ☒ | The exact sample size (*n*) for each experimental group/condition, given as a discrete number and unit of measurement |
| ☐ | ☒ | A statement on whether measurements were taken from distinct samples or whether the same sample was measured repeatedly |
| ☒ | ☐ | The statistical test(s) used AND whether they are one- or two-sided<br>*Only common tests should be described solely by name; describe more complex techniques in the Methods section.* |
| ☒ | ☐ | A description of all covariates tested |
| ☒ | ☐ | A description of any assumptions or corrections, such as tests of normality and adjustment for multiple comparisons |
| ☐ | ☒ | A full description of the statistical parameters including central tendency (e.g. means) or other basic estimates (e.g. regression coefficient) AND variation (e.g. standard deviation) or associated estimates of uncertainty (e.g. confidence intervals) |
| ☒ | ☐ | For null hypothesis testing, the test statistic (e.g. $F$, $t$, $r$) with confidence intervals, effect sizes, degrees of freedom and $P$ value noted<br>*Give P values as exact values whenever suitable.* |
| ☒ | ☐ | For Bayesian analysis, information on the choice of priors and Markov chain Monte Carlo settings |
| ☒ | ☐ | For hierarchical and complex designs, identification of the appropriate level for tests and full reporting of outcomes |
| ☒ | ☐ | Estimates of effect sizes (e.g. Cohen's $d$, Pearson's $r$), indicating how they were calculated |

*Our web collection on statistics for biologists contains articles on many of the points above.*

## Software and code

Policy information about availability of computer code

| | |
|---|---|
| Data collection | X-ray crystallographic data were collected at the European Synchrotron Radiation Facility (ESRF), Grenoble (France) at beamlines ID23-1 and ID30A |
| Data analysis | Data were processed with XDS and ccp4-implemented AIMLESS. The (Gt)RemA structure was determined by experimental phasing with Crank-2, manually built in Coot, and refined with PHENIX. Figures of protein structures were prepared with Pymol (www.pymol.org) and UCSF Chimera. |

For manuscripts utilizing custom algorithms or software that are central to the research but not yet described in published literature, software must be made available to editors and reviewers. We strongly encourage code deposition in a community repository (e.g. GitHub). See the Nature Portfolio guidelines for submitting code & software for further information.

## Data

Policy information about availability of data

All manuscripts must include a data availability statement. This statement should provide the following information, where applicable:

- Accession codes, unique identifiers, or web links for publicly available datasets
- A description of any restrictions on data availability
- For clinical datasets or third party data, please ensure that the statement adheres to our policy

Coordinates and structure factors of RemA crystal structures have been deposited and are now publicly available in the Protein Data Bank (PDB) with the accession codes 7BM2 (wildtype), 7BME (RemA-R18W) and 7P1W (RemA-R51A/R53A). Plasmids, primers and strains associated with this manuscript are available upon request to either G.B. or E.B.

# Field-specific reporting

Please select the one below that is the best fit for your research. If you are not sure, read the appropriate sections before making your selection.

[x] Life sciences [ ] Behavioural & social sciences [ ] Ecological, evolutionary & environmental sciences

For a reference copy of the document with all sections, see nature.com/documents/nr-reporting-summary-flat.pdf

# Life sciences study design

All studies must disclose on these points even when the disclosure is negative.

| | |
|---|---|
| Sample size | Experiments were performed in (at least) three biological replicates (see also: replication). |
| Data exclusions | no data were excluded |
| Replication | EMSAs were performed three times with independently purified charges of each protein, respectively.<br>Fig. 2f: LacZ-activity assays of ΔRemA (n=5), wild type RemA (n=9), RemA-R50A (n=3) and RemA-R51A (n=3).<br>Fig. 3c: LacZ promoter activity assays of ΔRemA (n=5), wild type RemA (n=9) and its variants carrying amino acid changes with helix1 RemA-P29S (n=3), RemA-R32A (n=3), RemA-D36S (n=3), RemA-D39A (n=3) and RemA-D36A/D39A (n=3).<br>Fig. 3h: LacZ promoter activity assays of ΔRemA (n=5), wild type RemA (n=9) and the RemA-R18W variant (n=4). |
| Randomization | The study was not randomized, because all experimental factors could be brought under direct experimental control. |
| Blinding | None of the experiments was blinded, because the experimentators had no expectations to a specific outcome of the experiment. |

# Reporting for specific materials, systems and methods

We require information from authors about some types of materials, experimental systems and methods used in many studies. Here, indicate whether each material, system or method listed is relevant to your study. If you are not sure if a list item applies to your research, read the appropriate section before selecting a response.

## Materials & experimental systems

| n/a | Involved in the study | |
|---|---|---|
| x | | Antibodies |
| x | | Eukaryotic cell lines |
| x | | Palaeontology and archaeology |
| x | | Animals and other organisms |
| x | | Human research participants |
| x | | Clinical data |
| x | | Dual use research of concern |

## Methods

| n/a | Involved in the study | |
|---|---|---|
| x | | ChIP-seq |
| x | | Flow cytometry |
| x | | MRI-based neuroimaging |
